# Cell-free circulating DNA integrity is an independent predictor of impending breast cancer recurrence

**DOI:** 10.18632/oncotarget.17384

**Published:** 2017-04-24

**Authors:** Jie Cheng, Katarina Cuk, Jörg Heil, Michael Golatta, Sarah Schott, Christof Sohn, Andreas Schneeweiss, Barbara Burwinkel, Harald Surowy

**Affiliations:** ^1^ Division of Molecular Epidemiology, German Cancer Research Center (DKFZ), Heidelberg, Germany; ^2^ Molecular Biology of Breast Cancer, Department of Gynecology and Obstetrics, University of Heidelberg, Heidelberg, Germany; ^3^ Department of Gynecology and Obstetrics, University Women's Clinic, Heidelberg, Germany; ^4^ National Center for Tumor Diseases, University of Heidelberg, Heidelberg, Germany

**Keywords:** breast cancer, recurrence, circulating DNA integrity, biomarker

## Abstract

Non-invasive blood-based molecule markers are evaluated as promising biomarkers these days. Here we investigated the potential of cell-free circulating DNA Integrity (cfDI) as blood-based marker for the prediction of recurrence during the follow-up of breast cancer patients within a prospective study cohort. cfDI was determined in plasma of 212 individuals, by measuring ALU and LINE1 repetitive DNA elements using quantitative PCR. A significant decrease of cfDI in recurrent breast cancer patients was observed. The group of patients who had impending recurrence during the follow-up had significant lower cfDI compared to the group of non-recurrent patients (P < 0.001 for ALU and LINE1 cfDI). cfDI could differentiate recurrent breast cancer patients from non-recurrent breast cancer subjects (area under the curve, AUC = 0.710 for ALU and 0.704 for LINE1). Univariate and multivariate analysis confirmed a significant association of recurrence and cfDI. Breast cancer patients with a lower cfDI had a much higher risk to develop recurrence than the patients with a higher cfDI (P = 0.020 for ALU cfDI and P = 0.019 for LINE1 cfDI, respectively). Further we show that cfDI is an independent predictor of breast cancer recurrence. In combination with other molecular markers, cfDI might be a useful biomarker for the prediction for breast cancer recurrence in clinic utility. We propose that cfDI might also be useful for the prediction of recurrence during the follow-up of other cancers.

## INTRODUCTION

Breast cancer is the most common female cancer, with more than 230,000 new cases estimated to be diagnosed in the United States in 2015 alone [1]. Several studies found that the average rates of recurrence were 11-30% at 5 years and 20-36.8% at 10 years after completion of initial treatments [2–5]. Breast cancer recurrence can be generally categorized into three types: local, regional and distant recurrence, whereas the first two are often combined into loco-regional recurrence [6, 7]. The 5-year PFS and OS were found to be 45% and 71% in the local recurrence group and 34% and 58% in the regional recurrence group [8–10].

To date researchers have identified several factors that are associated with breast cancer recurrence such as age, tumor size, focality, lymph node involvement, grade, estrogen receptor (ER) status, progesterone receptor (PR) status, human epidermal growth factor receptor 2 (HER2) status and Ki67 expression [11–14]. These factors are determined in the primary tumor and are obtained through traditional tumor biopsy [15]. However a tissue sample cannot be continuously monitored during therapy and follow-up of cancer patients [16].

Blood-based biomarkers hold great promises as they are easily accessible and reproducible [17]. A major advantage of blood-based biomarkers, including properties of the cfDNA, in the context of cancer recurrence is the fact that they can be monitored repeatedly, even after the primary tumor has been removed. In recent years blood-based biomarkers such as microRNAs, circulating tumor cells and others have been investigated for the diagnosis and prognosis of breast cancer [18–24]. Circulating DNA is described as cell-free DNA (cfDNA) or circulating tumor DNA present in serum or plasma [25, 26] and elevated cfDNA concentrations have been observed in some types of cancers [17]. The extent of cfDNA fragmentation has also been used in addition to cfDNA concentrations [27]. Generally, the cell free DNA integrity (cfDI) is calculated as the ratio of longer DNA fragments concentration to shorter ones from a specific genetic locus. cfDI has been studied as a biomarker for detection of some types of tumors like head and neck [28], breast cancer [29], renal cancer [30] and acute leukemia [31] et al. As cfDNA integrity can be monitored repeatedly even after the primary tumor has been removed, cfDI might provide the opportunity for an early detection of cancer relapse.

Here in this prospective nested study we aimed to investigate if cfDI can be a biomarker for predicting BC recurrence during the follow-up of BC patients after initial treatment.

## RESULTS

### Altered cfDI and cfDNA concentrations prior to breast cancer recurrence

**Figure 1:**
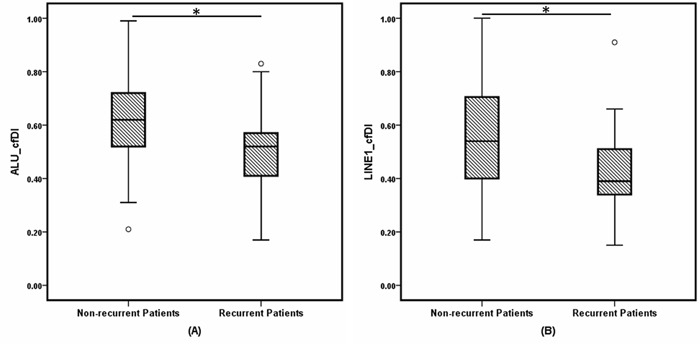
Box and whisker plots of cell-free DNA integrity (cfDI) in non-recurrent breast cancer patients and recurrent BC patients estimated from **(A)** ALU, **(B)** LINE1 targets. * indicates P less than 0.001.

**Figure 2:**
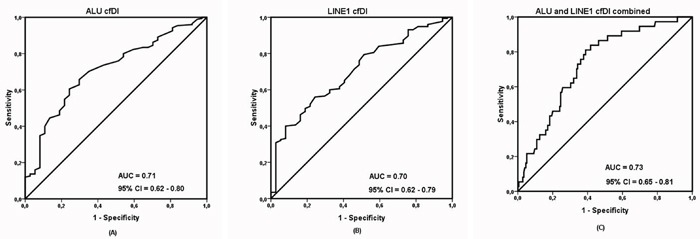
Receiver operating characteristic (ROC) analysis using **(A)** cell-free DNA integrity (cfDI) calculated from ALU targets, **(B)** cell-free DNA integrity calculated from LINE1 targets, **(C)** cfDI from ALU and LINE1 targets combined, to estimate the strength of the model to discriminate two groups, along with area under the curve (AUC) and 95% confidence interval (CI).

The results between the independently measured ALU and LINE1 elements were consistent with high correlation both for log_2_cfDNA concentration and cfDI (Supplementary Figure 1). As shown in Table 1 and Figure 1, patients with impending recurrence had a significantly lower cfDI (median ALU cfDI = 0.52, median LINE1 cfDI = 0.39) compared to the group of non-recurrent patients (median ALU cfDI =0.62, median LINE1 cfDI = 0.54) (P<0.0001 for each). In contrast, the concentration differences of both ALU and LINE1 were not significant between the two patient groups (ALU: P = 0.16; LINE1: P = 0.17) (Supplementary Figure 2). ROC analysis revealed that cfDI can distinguish patients with impending recurrence from non-recurrent patients with an AUC of 0.710 for ALU and 0.704 for LINE1 (Figure 2A, 2B). When ALU and LINE1 cfDI data were combined, discriminatory power with an AUC of 0.732 was reached (Figure 2C).

**Table 1:** Mean and median cfDI and cfDNA concentration of recurrent and non-recurrent groups calculated from ALU and LINE1 targets, and P-values of Wilcoxon rank sum tests comparing cfDI and log_2_cfDNA concentration between recurrent and non-recurrent breast cancer patients

Group	Index	Recurrent Patients		Non-Recurrent Patients		Comparison
		Mean ± SD	Median	Mean ± SD	Median	P-value
ALU	cfDI	0.51±0.14	0.52	0.62±0.16	0.62	**7.95E-05**
	cfDNA conc (ng/μl)	1.16±3.42	0.21	0.55±0.19	0.25	0.16
LINE1	cfDI	0.43±0.15	0.39	0.56±0.19	0.54	**1.11E-04**
	cfDNA conc (ng/μl)	1.43±4.26	0.26	0.53±0.40	0.33	0.17

Statistically significant P < 0.05 in the univariate and multivariate analysis is highlighted in bold.

*conc* concentration, SD standard deviation.

### Correlation of cfDI and cfDNA concentration with clinical characteristics

To investigate the influence of clinical factors on the detected cfDI and cfDNA concentrations in the samples, the associations between these measures and various clinical characteristics was calculated. Here, a true association was determined only if both ALU and LINE1 elements showed consistent results. As shown in Supplementary Table 1, age was the only factor which showed a consistent association on cfDNA concentration (P = 0.013 for ALU, P = 0.015 for LINE1), whereas it showed no significant association of cfDI. No associations with other factors were observed. Also, primary tumor parameters, including histological type, grading, tumor size, nodal status, ER status, PR status, HER2 status showed no influence on cfDI or cfDNA concentration. To test if cfDI may be affected by the time after therapy until blood withdrawal, we analyzed the correlation between cfDI and the time of the first follow-up date to the time of blood withdrawal which showed no correlation (P = 0.65 for ALU cfDI, P= 0.90 for LINE1 cfDI). What's more, we also found no significant difference of cfDI and cfDNA concentration of non-recurrent patients between this follow-up time to the follow-up time of the average recurrent patients (P > 0.1).

### Univariate and multivariate analysis of factors related to breast cancer recurrence

**Figure 3:**
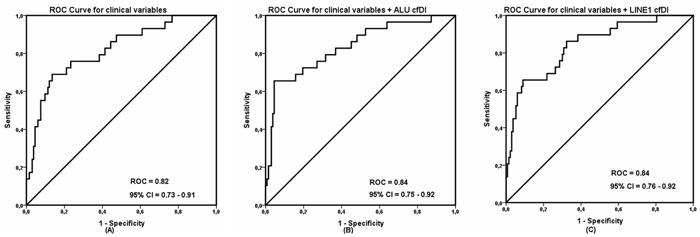
Receiver operating characteristic (ROC) analysis using **(A)** only clinical variables, **(B)** clinical variables with ALU cell-free DNA integrity and **(C)** clinical variables with LINE1 cell-free DNA integrity to estimate the strength of the model to discriminate two groups, along with area under the curve (AUC) and 95% confidence interval (CI).

Univariate analysis demonstrated that ALU and LINE1 cfDI, as well as primary tumor features such as tumor size, ER status, PR status, Ki67 expression level, and type of chemo-therapy were significantly associated with the recurrence status, as shown in Table 2. To evaluate if cfDI can predict breast cancer recurrence independent from the influence of these and other known factors related to recurrence [32], we performed multiple logistic regression analyses. The association of recurrence and cfDI remained significant (P = 0.020 and 0.019 for ALU and LINE1, respectively) with an odds ratio for developing recurrence of 3.69 (95% CI 1.23 – 11.02) for ALU cfDI and 3.74 (95% CI 1.24 – 11.27) for LINE1 cfDI. By using the highest cfDI quartile (Q4) as reference category in the interquartile analysis, it was shown that the risk for patients to develop BC recurrence significantly (P for trend = 0.011 for ALU and P for trend = 0.016 for LINE1) increased for patients in lower cfDI quartiles (Q3, Q2, Q1) compared to patients in the highest cfDI quartile, with an OR between the lowest and highest quartiles of 5.8 (95% CI 1.8 – 18.7) for ALU and10.9 (95% CI 2.4-50.7) for LINE1, as shown in Table 3 and 4. Finally, we constructed different multivariate models to investigate the prognostic ability of cfDI when added with clinical variables. In this way we calculated the corresponding area under the ROC curve was 0.82 (95% CI = 0.73 – 0.91) for clinical variables alone. When combined with cfDI, AUC was increased to 0.84 (95% CI = 0.75 – 0.92) for ALU cfDI and 0.84 (95% CI = 0.76 – 0.92) for LINE1 cfDI (Figure 3). Taken together, these observations confirmed that a decreased cfDI is associated with an increased risk of impending breast cancer recurrence and provide evidence that cfDI is an independent predictor BC recurrence.

**Table 2:** Univariate and multivariate analysis of factors related to recurrence in breast cancer patients

Variables				ALU			LINE1		
	univariate analysis			multivariate analysis			multivariate analysis		
	P value	OR	95%CI	P value	OR	95%CI	P value	OR	95%CI
LINE1 cfDI	**8.45E-04**	3.96	(1.77-8.88)				**0.019**	3.74	(1.24-11.27)
ALU cfDI	**5.63E-04**	4.15	(1.85-9.31)	**0.020**	3.69	(1.23-11.02)			
Age	0.58	1.01	(0.98-1.04)	0.65	0.99	(0.94-1.04)	0.67	0.99	(0.94-1.04)
Histotype	0.46	0.84	(0.54-1.32)	0.24	0.68	(0.35-1.23)	0.25	0.67	(0.34-1.32)
Grading	0.059	0.63	(0.39-1.02)	0.47	0.70	(0.27-1.84)	0.49	0.72	(0.29-1.80)
Focal	0.70	1.20	(0.49-2.94)	0.080	3.52	(0.86-14.37)	**0.042**	4.32	(1.06-17.68)
Tumor Size	**7.21E-3**	0.49	(0.29-0.83)	0.084	0.38	(0.13-1.14)	0.082	0.42	(0.16-1.12)
Nodus Stages	0.039	0.47	(0.23-0.96)	0.11	0.38	(0.12-1.24)	0.063	0.32	(0.099-1.06)
ER	**6.15E-03**	3.13	(1.38-7.08)	0.056	6.38	(0.95-42.71)	0.078	5.28	(0.83-33.71)
PR	**0.013**	1.83	(0.84-3.99)	0.25	0.35	(0.060-2.07)	0.28	0.39	(0.073-2.12)
HER2	0.69	1.26	(0.41-3.90)	0.44	2.50	(0.25-24.92)	0.38	2.88	(0.28-29.89)
p53	0.53	1.01	(0.99-1.02)	**0.044**	1.02	(1.00-1.05)	0.097	1.02	(1.00-1.04)
Ki67	0.066	0.98	(0.97-1.00)	0.22	0.98	(0.96-1.01)	0.13	0.98	(0.95-1.01)
Operation	0.76	0.90	(0.44-1.84)	0.36	0.56	(0.16-1.96)	0.35	0.55	(0.16-1.92)
Radio_therapy	0.54	1.34	(0.53-3.36)	0.32	2.23	(0.45-10.99)	0.28	2.33	(0.50-10.84)
Chemo_therapy	0.068	0.47	(0.21-1.06)	0.13	0.37	(0.099-1.34)	0.24	0.46	(0.124-1.69)

Statistically significant P < 0.05 in the univariate and multivariate analysis is highlighted in bold.

Abbreviations: OR - odds ratio, CI - confidence interval.

**Table 3:** Association of ALU cfDI with recurrence of breast cancer

ALU	Non-Recurrent Patients	Recurrent Patients	OR	95%CI
0.17-0.50 (Quartile 1)	36	16	5.8	(1.8-18.7)
0.50-0.59 (Quartile 2)	41	11	3.5	(1.0-11.8)
0.59-0.71 (Quartile 3)	46	6	1.7	(0.5-6.4)
0.71-0.99 (Quartile 4)	52	4	1.00 (reference)	
P for trend	**0.011**			

Statistically significant P < 0.05 in the univariate and multivariate analysis is highlighted in bold.

Abbreviations: OR - odds ratio, CI - confidence interval.

**Table 4:** Association of LINE1 cfDI with recurrence of breast cancer

LINE1	Non-Recurrent Patients	Recurrent Patients	OR	95%CI
0.15-0.38 (Quartile 1)	37	15	10.9	(2.4-50.7)
0.38-0.52 (Quartile 2)	41	12	7.9	(1.7-37.3)
0.52-0.67 (Quartile 3)	43	8	5.0	(1.0-24.9)
0.67-0.99 (Quartile 4)	54	2	1.00 (reference)	
P for trend	**0.016**			

Statistically significant P < 0.05 in the univariate and multivariate analysis is highlighted in bold.

Abbreviations: OR - odds ratio, CI - confidence interval.

## DISCUSSION

By using a prospective nested study design, we identified that cfDI can be an independent predictor of impending breast cancer recurrence during the follow-up of breast cancer cases. To our knowledge, this is the first study that investigates plasma cfDI as an independent marker for prediction of breast cancer recurrence.

We observed significant decrease of cfDI in the group of patients who had impending recurrence compared to the group of non-recurrent patients (P < 0.001 for ALU and LINE1). The area under the curve (AUC) for ALU and LINE1 is 0.710 and 0.704 indicates that cfDI has moderate ability to differentiate recurrent breast cancer patients from non-recurrent breast cancer subjects. What is more, multivariate analysis confirmed a significant association of recurrence and cfDI (OR = 3.69 for ALU and 3.74 for LINE1). cfDI can also improve the discrimination ability when combined with clinical variables.

The investigation of circulating molecular markers in peripheral blood (“liquid biopsies”) is of great importance because of the advantages such as easily accessible, reproducible and early detectable in cancer [20]. Many biomarkers like microRNA, circulating DNA, circulating tumor cells are now being investigated as markers of diagnosis or prognosis in different types of cancer and these markers show great potential in the clinical utility [33–36]. Among these, cell-free circulating DNA integrity (cfDI) is becoming a potential biomarker for cancer diagnosis and prognosis in recent years.

Several studies have identified an altered integrity of cfDNA in malignant cancer patients compared to healthy controls. However these studies are heterogeneous with some studies showed a reduced cfDI in malignant cancer patients [6, 37–39], while others reported an increased cfDI [27, 28, 40, 41]. Many hypotheses have been applied to explain it. With direct visualization by gel electrophoresis, Giacona et al found that cfDNA from healthy individuals had three to five fold multiples of nucleosome-associated DNA length, and longer fragments compared to cfDNA in pancreatic cancer patients [42]. By massively parallel sequencing, Jiang found elevated amounts of shorter mitochondrial DNA molecules in plasma of carcinoma patients compared to healthy subjects [43]. On the other hand, it was hypothesized that in healthy controls cfDNA fragments are mainly released by apoptotic cells while in cfDNA in cancer patients they are thought to be predominantly released by malignant cells undergoing different pathophysiological processes including necrosis, autophagy, or mitotic catastrophe and thus show increased lengths [44]. However this explanation has not been supported by experimental proof so far, and necrotic DNA has been shown to account for increased cfDI in only a small fraction of cancer patients [45].

As the most abundant classes of repetitive DNA elements, ALU and LINE1 cover approximately 10% and 17% of the genome [46]. While the concentration of cfDNA can vary across several orders of magnitude [47], the cfDI as a ratio of nested long and short cfDNA fragment concentrations obtains standardized values between 0 and 1 which can be observed even in low cfDNA concentration. In addition, these measures reflect a global status of the circulating DNA rather than only one specific genetic locus. In this study, we observed a very good correlation from results between ALU and LINE1. Our concordant observations from independent cfDI measurements of these two elements minimize the possibility of false-positive results.

Many studies have identified the screening or diagnositic relevance of cfDI. However, few focus on the prognostic value of cfDI in cancer. Umetani et al measured serum DNA integrity in 51 healthy women and 83 patients with primary breast cancer by ALU-PCR and observed that cfDI was related to breast cancer AJCC (American Joint Committee on Cancer) stages [41]. It showed that serum DNA integrity was the predictor of lymph nodes metastasis by multivariate logistic analysis. Nevertheless, it only involved in lymph nodes metastasis. In another study about cfDI and its prognostic value in breast cancer, Iqbal et al compared DNA integrity in 25 relapsed and 61 non-relapsed patient samples and observed a significant difference between two groups (P = 0.005) [48]. DNA integrity also has significant association with disease free survival in breast cancer. Here we included all the types of recurrence including local, regional and distant metastases to observe whether cfDI can be a predictable biomarker for recurrence.

As the promising non-invasive biomarkers of clinic utility, it is of great importance to apply a standardized sample processing protocol. Firstly we prefer plasma to serum in cfDI study since studies have found the coagulation process in serum which would induce high variability of the spectrum of circulating nucleic acids [49]. In our study, we conducted a standardized protocol which has been validated in the other study [6]. Furthermore, for a specific gene the correct and validated primer design is essential. Besides its origin, factors for example time between sample collection and processing, plasma purification, the number of freeze-thaw cycles and the employed cfDNA extraction methods can affect cfDNA quality and quantity and thus cfDI measurements [50, 51]. We employed a standardized protocol that included sample processing within 2 hours of blood collection and a two-step centrifugation for plasma purification with a first step at 1300g for 20 min to separate the plasma from blood cells, followed by a second centrifugation step at 15500g for 10 min. This high-speed centrifugation of plasma removes cell debris and prevents interference of contaminating cellular DNA in cfDI measurements [6]. Prior to extraction, only one freeze-thaw cycle was ensured for all plasma samples.

In our study, subjects were included only if the patients has survival at least six months after treatment of the primary breast cancer, to avoid effects of surgery or chemotherapy on the cfDI. We did not observe an influence of the time period between the last time of therapy and blood withdrawal on the cfDI. Characteristics of the primary tumor, for example age, tumor size, focality, nodal status, hormone receptor status are known to be associated with breast cancer recurrence [12]. Likewise, here significant differences were observed in tumor size, ER, PR and p53 between non-recurrent and recurrent patients. Using binary logistic regression, we identified cfDI as an independent biomarker associated with recurrence.

Limitation of this study is the small sample size of the study however it is higher than the size of many other cfDI-related studies. It is not possible for us to compare in different subgroups in this study. Meanwhile the unbalanced number of recurrent and non-recurrent patients impedes the statistical power of this study. Large sample size studies are needed to confirm the results. Due to the lack of prospective studies with standardized processed sample material as mentioned above in other studies, our study is the first study of cfDI in plasma as a predictor in breast cancer recurrence so far. Nevertheless, it would be worthwhile to investigate cfDI combined with other blood-based biomarker in further prospective studies with excellent plasma material. Further it will be interesting to evaluate if cfDI can also contribute to the prediction of recurrence during disease follow-up in other cancers.

In summary, our study shows that cfDI can be an independent predictor of recurrence in breast cancer patients and might be a valuable marker as part of a molecular, blood-based multi-marker assay.

## MATERIALS AND METHODS

### Study subjects

The GENOM study was approved by the Ethical Committee of the University of Heidelberg (Heidelberg, Germany). Breast cancer patients with a primary tumor diagnosed between November 2008 and July 2015 were included. All subjects were females and Caucasians. For each patient blood samples were collected during follow-up visits in intervals of six months after initial therapy of the primary breast cancer. Recurrent patients were included if collected plasma samples from follow-up time points were available that fulfilled the following criteria: (1) the sample was taken 0-9 months before diagnosis of the recurrence and (2) the recurrence did not occur within the one year after initial therapy. Non-recurrent patients were included for plasma samples at FU3 which had been taken after at least 1 year of follow-up after therapy and at least 18 months of known, recurrence-free follow-up after sample collection. The clinical flow diagram for sample chosen was shown in Supplementary Figure 3. In total, 175 non-recurrent patients and 37 recurrent patients were included in this study. All patients’ demographic and clinical data are presented in Table 5.

**Table 5:** Distribution of clinical features of patients used in circulating DNA analyses

Characteristics		Recurrent Patients		Non-Recurrent Patients	
		n=37	(%)	n=175	(%)
Age	Mean	55.5		56.8	
	Median	55		55	
	Range	32-80		28-81	
Menopausal status	Pre	12	(32.4%)	58	(33.1%)
	Peri	1	(2.7%)	10	(5.7%)
	Post	24	(64.8%)	105	(60%)
	NA	0	(0.0%)	2	(1.1%)
Histology	IDC	30	(81.1%)	151	(86.3%)
	ILC	4	(10.8%)	18	(10.3%)
	DCIS	1	(2.7%)	5	(2.9%)
	NA	2	(5.4%)	1	(2.7%)
Grading	1	1	(2.7%)	21	(14.3%)
	2	18	(48.6%)	111	(60.9%)
	3	17	(45.9%)	34	(18.0%)
	NA	1	(2.7%)	9	(6.8%)
Focality	Uni	29	(78.4%)	135	(75.2%)
	Multi	7	(18.9%)	39	(24.1%)
	NA	1	(2.7%)	1	(0.8%)
Tumor Size	Tis	2	(5.4%)	5	(2.8%)
	T0	2	(5.4%)	15	(8.6%)
	T1	11	(29.7%)	76	(43.4%)
	T2	14	(37.8%)	67	(38.3%)
	T3	4	(10.8%)	9	(5.1%)
	T4	4	(10.8%)	2	(5.4%)
Lymph node	N0	21	(56.8%)	121	(69.1%)
	N1	10	(27.0%)	33	(18.9%)
	N2	1	(2.7%)	12	(6.9%)
	N3	5	(13.5%)	9	(5.1%)
ER status	Positive	25	(67.6%)	150	(87.2%)
	Negative	12	(32.4%)	23	(11.3%)
	NA	0	(0.0%)	2	(1.5%)
PR Status	Positive	25	(67.6%)	137	(82.0%)
	Negative	12	(32.4%)	36	(16.5%)
	NA	0	(0.0%)	2	(1.5%)
HER2 Status	Positive	4	(10.8%)	23	(3.8%)
	Negative	32	(86.5%)	146	(91.7%)
	NA	1	(2.7%)	6	(4.5%)
p53 Score	0-1	15	(40.5%)	54	(30.9%)
	2-10	10	(27.0%)	54	(30.9%)
	> 10	6	(16.2%)	34	(19.4%)
	NA	6	(16.2%)	33	(18.9%)
Ki67 Score	1-10	6	(16.2%)	79	(45.1%)
	11-20	8	(21.6%)	33	(18.9%)
	21-50	11	(30.4%)	33	(18.9%)
	> 50	10	(29.7%)	22	(12.6%)
	NA	2	(5.4%)	8	(4.6%)
Chemo_therapy	Yes	28	(75.7%)	104	(57.9%)
	No	9	(24.3%)	71	(42.1%)
Radio_therapy	Yes	30	(81.1%)	149	(86.5%)
	No	7	(18.9%)	26	(13.5%)
Endocrine_therapy	Yes	24	(64.9%)	117	(63.2%)
	No	13	(35.1%)	58	(36.8%)
Surgical Type	BCT	25	(67.6%)	132	(78.9%)
	Mastectomy	10	(32.4%)	42	(21.1%)
	NA	2	(32.4%)	1	(21.1%)
Distant Recurrence No.	27	lung	8		
		liver	13		
		bone	20		
		other	15		
Local-regional Recurrence No.	10	local	7		
		regional	3		

Abbreviations: ER - oestrogen receptor, PR - progesterone receptor, HER2 - human epidermal growth factor 2, T - tumor size, N - lymph node status, BCT - breast conserving therapy.

### Sample collection and cfDNA extraction

Peripheral blood was collected from all patients in 9 ml EDTA tubes (S-Monovette R, Sarstedt, Nümbrecht, Germany). Blood was centrifuged at 1300g for 20 min at 10°C within two hours of blood withdrawal. The supernatant was transferred and centrifuged at 15500g for 10 min at 10°C. This step was done to make sure that the plasma was free of cells or cell debris. The plasma supernatant was snap frozen in liquid nitrogen and stored at -80°C until further use. cfDNA was extracted from 800μl plasma using the QIAamp DNA Blood Mini Kit (Qiagen, Hilden, Germany) with minor modifications as described before [6]. Extracted cfDNA was eluted in 30 μl of AE elution buffer. The eluate was re-applied onto the column, and the final eluate was collected and stored at -20°C. Blood samples from recurrent and non-recurrent patients were extracted together to avoid batch effects.

### Measurement of cfDI and cfDNA concentration

Concentration and integrity of circulating DNA in blood plasma were analyzed by measuring the abundances of short and long fragments from two repetitive DNA elements, ALU (ALU-111bp, ALU-260bp) and LINE1 ( LINE1-97bp, LINE1-266bp) as described before [6]. All primer sequences and amplicon lengths are given in Supplementary Table 2. The fragment concentrations were measured in triplicates by quantitative PCR using ABsolute qPCR SYBR Green Mix (Thermo Scientific, Carlsbad, USA) and the LightCycler480 system (Roche Diagnostics, Mannheim, Germany). The cfDNA eluate was diluted 1:20 before use to achieve optimal PCR efficiency. Concentrations of the long and short fragments were calculated using the absolute quantification method according to the Light Cylcer 480 software instructions. cfDI was calculated as the ratio of long divided by short fragments concentrations for each of the elements: ALU-260/111, LINE1-266/97 as described before [6]. As short fragments were nested within the long fragments, cfDI ranged from 0 to 1. Short fragment concentrations were regarded as overall cfDNA concentrations.

### Statistical analysis

All statistical analyses were carried out using the PASW Statistics 18.0 (SPSS, Chicago, IL, USA) package. cfDNA concentrations are not normally distributed and thus were log_2_-transformed for all data analysis. Differences of cfDNA concentrations and cfDI between the sample groups were evaluated with the Mann-Whitney U test. Influences of clinical parameters on cfDI and cfDNA concentration were studied by Mann-Whitney U test (for categorical and binary data), Spearman correlation permutation tests (for quantitative and continuous data), Jonckheere-Terpstra tests (for ordinal data) and Kruskal-Wallis H tests (for dependent ordinal data). Receiver operating characteristic (ROC) analysis was carried out to assess the discriminatory power of cfDI and cfDNA concentration between non-recurrent and recurrent groups and the corresponding area under the curve (AUC) was calculated. Univariate logistic regression was used to compare different variables between the sample groups. Multivariate logistic regression analyses based on all women grouped by recurrence status were performed to estimate the odds ratio and 95% confidence interval (CI), adjusting for known variables associated with recurrence, such as tumor size, lymph node status, histological grade as well as for significant variables in the univariate analysis. Interquartile analysis of cfDI and recurrence of breast cancer was conducted by logistic regression, with the highest cfDI quartile taken as the reference. P-values less than 0.05 were considered as statistically significant.

## SUPPLEMENTARY MATERIALS FIGURES AND TABLES


