# Supplementary material for: Cell-free circulating DNA integrity is an independent predictor of impending breast cancer recurrence
Source: Oncotarget. 2017 Apr 24;8(33):54537–47. doi: 10.18632/oncotarget.17384 (PMC5589601; doi:10.18632/oncotarget.17384)
Supplement: Supplementary file 1 [file oncotarget-08-54537-s001.pdf]

Cell-free circulating DNA integrity is an independent predictor of impending breast cancer recurrence

SUPPLEMENTARY FIGURES AND TABLES

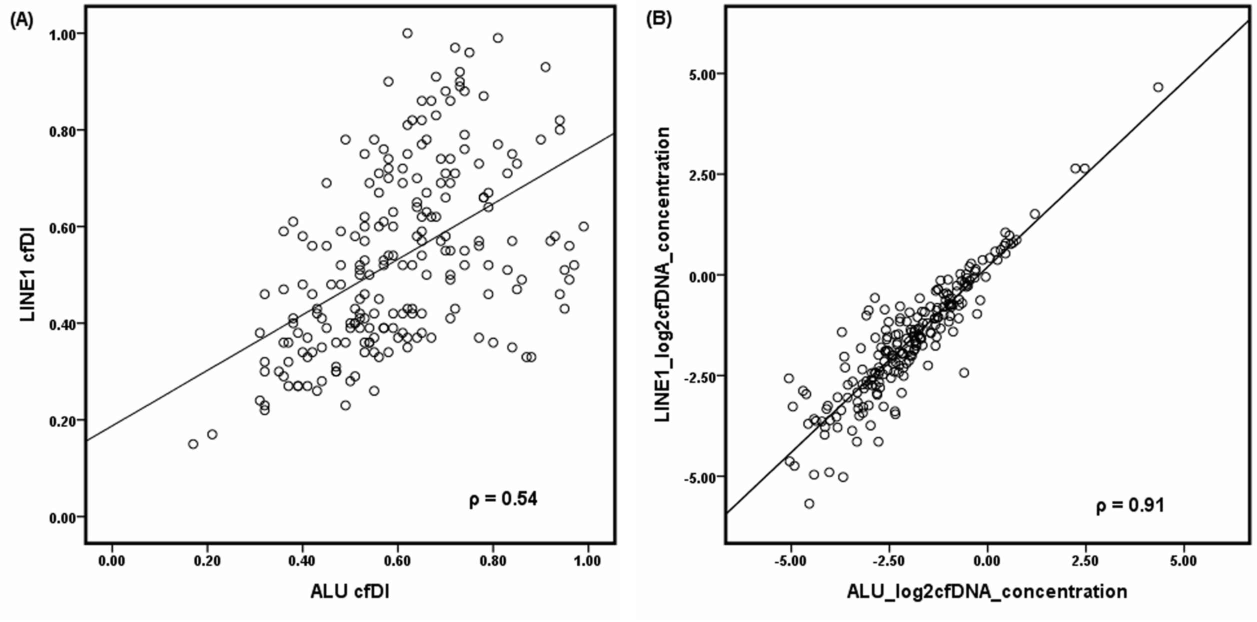

Supplementary Figure 1: Correlation of **(A)** cell-free DNA integrity (cfDI) and **(B)** cell-free DNA (cfDNA) concentration estimated from ALU and LINE1 repetitive elements.

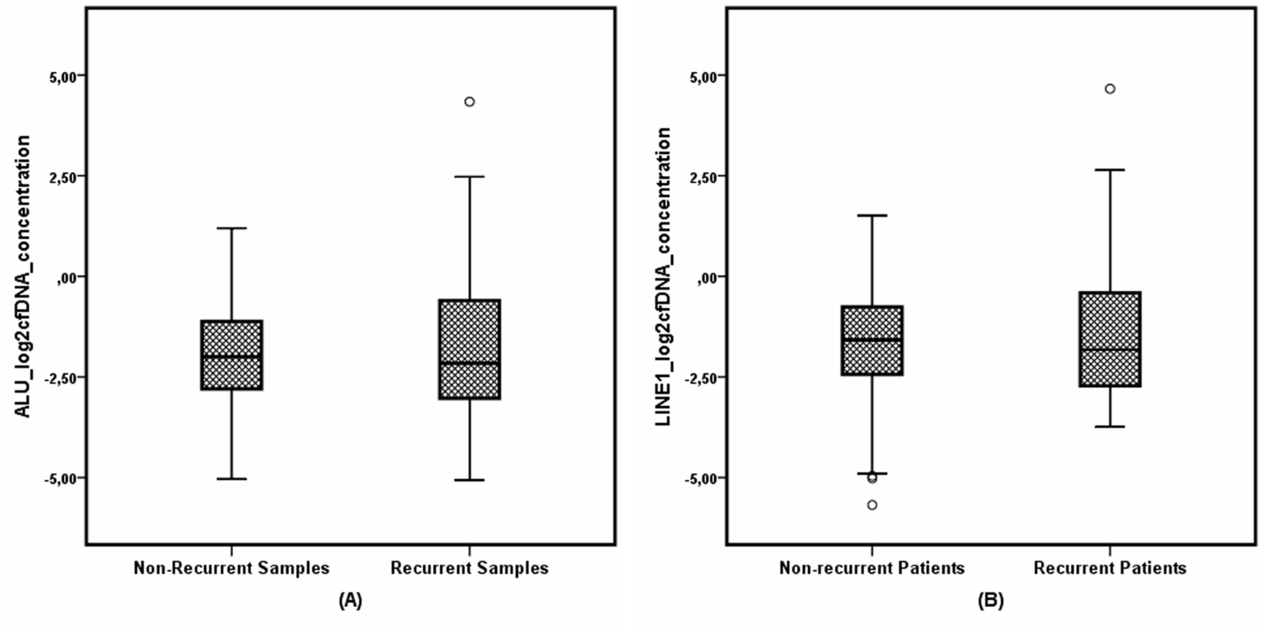

Supplementary Figure 2: Box and whisker plots of cell-free DNA concentration in non-recurrent and recurrent BC patients estimated from **(A)** ALU and **(B)** LINE1 targets.

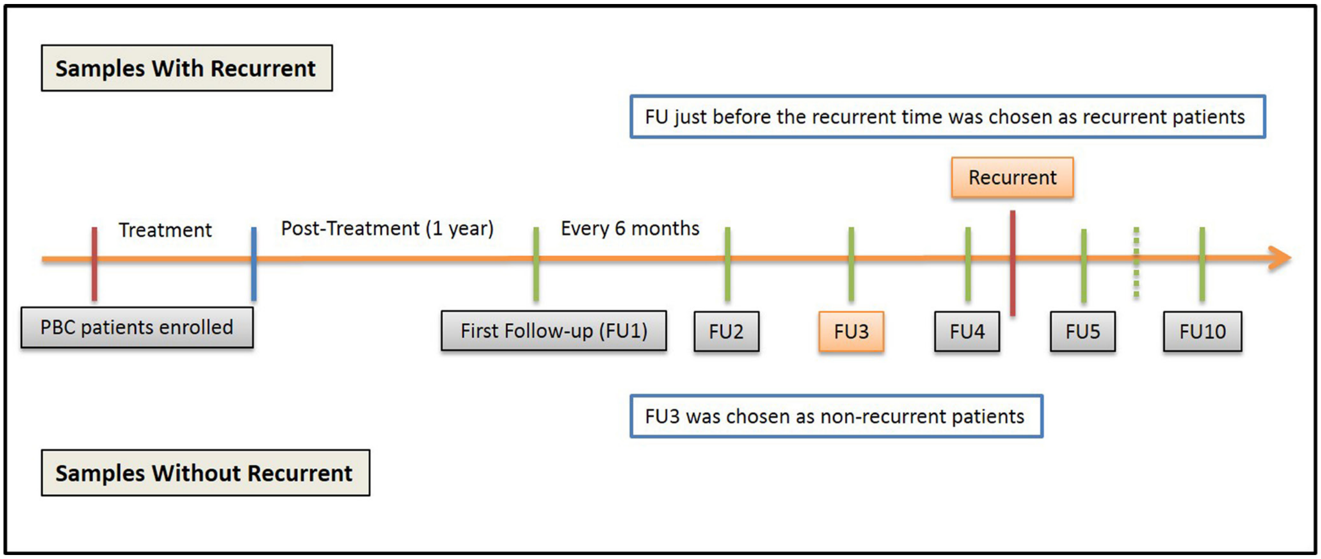

Supplementary Figure 3: The process flow diagram of selected samples.

Supplementary Table 1: Correlation of cfDI and log_2cfDNA concentration to clinical features in all patients

Correlation	Denotation of results.	ALU log_2DNAconc		ALU cfDI		LINE1 log_2DNAconc		LINE1 cfDI	
		Result	P	Result	P	Result	P	Result	P
Age[a]	ρ value	0.171	**0.013**	-0.053	0.446	0.168	**0.015**	0.006	0.930
Menopause[b]	ρ value	0.121	0.162	-0.073	0.312	0.137	0.100	-0.037	0.457
Histotype[d]	ρ value	-0.001	0.516	0.046	0.628	-0.006	0.733	-0.003	0.844
Grading[b]	ρ value	-0.053	0.500	-0.106	0.075	-0.032	0.533	-0.166	**0.020**
Fokal[c]	uni-multifocal	0.077	0.269	0.097	0.162	0.090	0.194	0.051	0.466
T[c]	Tis,0,1 vs T2,3,4	0.010	0.885	-0.036	0.606	0.010	0.089	-0.009	0.896
N[c]	postive-negative	0.021	0.760	-0.066	0.341	-0.017	0.801	-0.007	0.925
ER[c]	positive-negative	0.099	0.153	0.056	0.419	0.068	0.323	0.083	0.227
PR[c]	positive-negative	0.095	0.183	0.105	0.141	0.089	0.214	0.093	0.195
HER2[c]	positive-negative	-0.127	0.071	0.034	0.627	-0.198	**0.004**	0.019	0.787
p53[a]	ρ value	0.074	0.334	-0.146	0.056	0.069	0.365	-0.019	0.804
Ki67[a]	ρ value	-0.038	0.579	-0.071	0.301	-0.053	0.444	-0.132	0.055
OP[c]	BCT-Mastectomy	-0.178	0.396	0.085	0.612	-0.270	0.363	-0.025	0.443
Radiotherapy[c]	with-without therapy	0.101	0.141	-0.059	0.390	0.106	0.124	0.054	0.435
Endotherapy[c]	with-without therapy	-0.044	0.522	0.003	0.971	-0.101	0.141	-0.013	0.856
Chemotherapy[c]	with-without therapy	0.013	0.851	-0.038	0.584	0.028	0.684	-0.127	0.065

P< 0.05 is highlighted in bold. Tests performed are[a] Spearman correlation permutation test, [b] Jonckheere-Terpstra test, and [c] Wilcoxon rank sum test and [d] Kruskal-Wallis H test, depending on the type of variable. Statistically significant p<0.05 is indicated in bold.

Abbreviations: cfDI - cell-free DNA integrity, cfDNA - cell-free DNA, conc - concentration, ER –oestrogen receptor, PR - progesterone receptor, HER2 - human epidermal growth factor 2, T- tumor size, N - lymph node status, OP – operation, BCT – breast conserving therapy.

Supplementary Table 2: Primer sequences of short and long fragments amplified from ALU and LINE1 elements

	Forward Primer	Reverse Primer	Amplicon Size (bp)	Ta(°C)
ALU-111	CTGGCCAACATGGTGAAAC	AGCGATTCTCCTGCCTCAG	111	61
ALU-260	ACGCCTGTAATCCCAGCA	CGGAGTCTCGCTCTGTCG	260	61
LINE1-97	TGGCACATATACACCATGGAA	TGAGAATGATGGTTTCCAATTTC	97	65
LINE1-266	ACTTGGAACCAACCCAAATG	CACCACAGTCCCCAGAGTG	266	65

Abbreviations: bp – base pair, Ta - annealing temperature.
